# Enhancement of astrocytic gap junctions Connexin43 coupling can improve long‐term isoflurane anesthesia–mediated brain network abnormalities and cognitive impairment

**DOI:** 10.1111/cns.13974

**Published:** 2022-09-25

**Authors:** Rui Dong, Pin Lv, Yuqiang Han, Linhao Jiang, Zimo Wang, Liangyu Peng, Zhengliang Ma, Tianjiao Xia, Bing Zhang, Xiaoping Gu

**Affiliations:** ^1^ Department of Anesthesiology The Affiliated Drum Tower Hospital of Nanjing University Medical School Nanjing China; ^2^ Department of Radiology The Affiliated Drum Tower Hospital of Nanjing University Medical School Nanjing China; ^3^ Medical School Nanjing University Nanjing China; ^4^ Jiangsu Key Laboratory of Molecular Medicine Nanjing China; ^5^ Institute of Medical Imaging and Artificial Intelligence Nanjing University Nanjing China; ^6^ Institute of Brain Science Nanjing University Nanjing China

**Keywords:** astrocytic network, cognitive impairment, Connexin43, gap junction, isoflurane, MEMRI, rs‐fMRI

## Abstract

**Aim:**

Astrocytes are connected by gap junctions Connexin43 (GJs‐Cx43) forming an extensive intercellular network and maintain brain homeostasis. Perioperative neurocognitive disorder (PND) occurs frequently after anesthesia/surgery and worsens patient outcome, but the neural circuit mechanisms remain unclear. This study aimed to determine the effects of the GJs‐Cx43–mediated astrocytic network on PND and ascertain the underlying neural circuit mechanism.

**Methods:**

Male C57BL/6 mice were treated with long‐term isoflurane exposure to construct a mouse model of PND. We also exposed primary mouse astrocytes to long‐term isoflurane exposure to simulate the conditions of in vivo cognitive dysfunction. Behavioral tests were performed using the Y‐maze and fear conditioning (FC) tests. Manganese‐enhanced magnetic resonance imaging (MEMRI) and resting‐state functional magnetic resonance imaging (rs‐fMRI) were used to investigate brain activity and functional connectivity. Western blot and flow cytometry analysis were used to assess protein expression.

**Results:**

Reconfiguring the astrocytic network by increasing GJs‐Cx43 expression can modulate 22 subregions affected by PND in three ways: reversed activation, reversed inhibition, and intensified activation. The brain functional connectivity analysis further suggests that PND is a brain network disorder that includes sleep‐wake rhythm–related brain regions, contextual and fear memory–related subregions, the hippocampal‐amygdala circuit, the septo‐hippocampal circuit, and the entorhinal‐hippocampal circuit. Notably, remodeling the astrocytic network by upregulation of GJs‐Cx43 can partially reverse the abnormalities in the above circuits. Pathophysiological degeneration in hippocampus is one of the primary hallmarks of PND pathology, and long‐term isoflurane anesthesia contributes to oxidative stress and neuroinflammation in the hippocampus. However, promoting the formation of GJs‐Cx43 ameliorated cognitive dysfunction induced by long‐term isoflurane anesthesia through the attenuation of oxidative stress in hippocampus.

**Conclusion:**

Enhancing GJs‐Cx43 coupling can improve brain network abnormalities and cognitive impairment induced by long‐term isoflurane anesthesia, its mechanisms might be associated with the regulation of oxidative stress and neuroinflammation.

## Graphical abstract

Perioperative neurocognitive disorder (PND) is a brain network disorder that includes sleep‐wake rhythm–related brain regions, contextual and fear memory–related subregions, the hippocampal‐amygdala circuit, the septo‐hippocampal circuit, and the entorhinal‐hippocampal circuit. Increasing gap junctions Connexin43 (GJs‐Cx43) coupling could ameliorate the abnormal brain function network patterns induced by long‐term isoflurane anesthesia and contribute to the underlying neural circuits associated with PND.

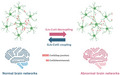

## 1 INTRODUCTION

Perioperative neurocognitive disorder (PND) is a major complication characterized by impaired memory, learning, and executive function in the short‐ and long term after surgery and anesthesia, which causes disability and distress for millions of patients annually.^1^ The specific molecular mechanisms contributing to PND pathogenesis remain poorly understood at present. Anesthetics should, in theory, produce a temporary and reversible loss of consciousness and reactivity without any additional side effects. However, isoflurane has both acute and chronic effects on brain function,^2^, ^3^ and prolonged isoflurane exposure could lead to cognitive dysfunction.^4^, ^5^, ^6^, ^7^ Notably, with the global spread of the coronavirus disease 2019 (COVID‐19) pandemic, there has been a severe shortage of intravenous sedatives for critically ill COVID‐19 patients.^8^ Many hospitals have turned to inhaled anesthesia as an alternative to intravenous sedation because it has shown benefits in animals and patients with acute respiratory distress syndrome.^8^, ^9^ Among the available inhaled sedative drugs, isoflurane offers the greatest potency with the lowest dosing requirements for COVID‐19 patients.^8^ Therefore, based on this realistic problem, we constructed a mouse PND model through long‐term isoflurane anesthesia to explore its related pathophysiological mechanisms.

Astrocytes in the central nervous system (CNS) form interconnected networks coupled with gap junctions (GJs), which are primarily composed of the channel protein Connexin43 (Cx43).^10^ Astrocytes regulate neural activity and maintain homeostasis by sensing the activity of neurons through these networks. The process by which one GJ breaks and forms two Cx43 molecules is called GJs‐Cx43 decoupling. Our previous studies showed that long‐term isoflurane exposure causes GJs‐Cx43 decoupling, resulting in cognitive dysfunction.^11^ However, the effects of GJs‐Cx43 decoupling on brain functional networks and structure remain unclear.

Previous research in PND has primarily focused on the microscale or syncytium scale, and mesoscale studies are rare. The neuroimaging technique offers a way of visualizing changes in brain function in vivo with high resolution at the mesoscale. Resting‐state functional magnetic resonance imaging (rs‐fMRI) determines functional connectivity (FC) across brain regions by measuring temporal correlations of blood oxygenation level–dependent signals to reflect communication between different brain areas.^12^ Furthermore, many studies have suggested that brain function arises from the concerted activity of a neuron‐astrocyte network.^13^, ^14^

As a biological analog of calcium ions (Ca2^+^), manganese ions (Mn^2+^) can enter neural cells via voltage‐gated calcium channels and be transported to adjacent cells during depolarization^15^; therefore, active neural circuits can be detected by tracking the transport of Mn^2+^ in the brain.^16^ In addition, unlike Ca^2+^, Mn^2+^ is paramagnetic and shortens the proton relaxation time constant (T1) in tissues where it accumulates, which enhances the signals in these areas on T1‐weighted (T1WI) magnetic resonance imaging (MRI) scans. In the brain, manganese enters both neurons and astrocytes, whereas 80% of the cerebral Mn^2+^ accumulates in astrocytes.^17^ Importantly, manganese‐enhanced MRI (MEMRI) has been proven to investigate astrocyte‐neuron interactions through quantification of brain Cx43 functional activity,^18^ which helps us to determine whether alterations in Cx43 conformation cause different patterns of brain activation. Here, the information on the brain regions of PND and brain alterations associated with GJs‐Cx43 were investigated through the combination methods of molecular biology, MEMRI, and rs‐fMRI.

## 2 MATERIALS AND METHODS

### 2.1 Animals

**FIGURE 1:**
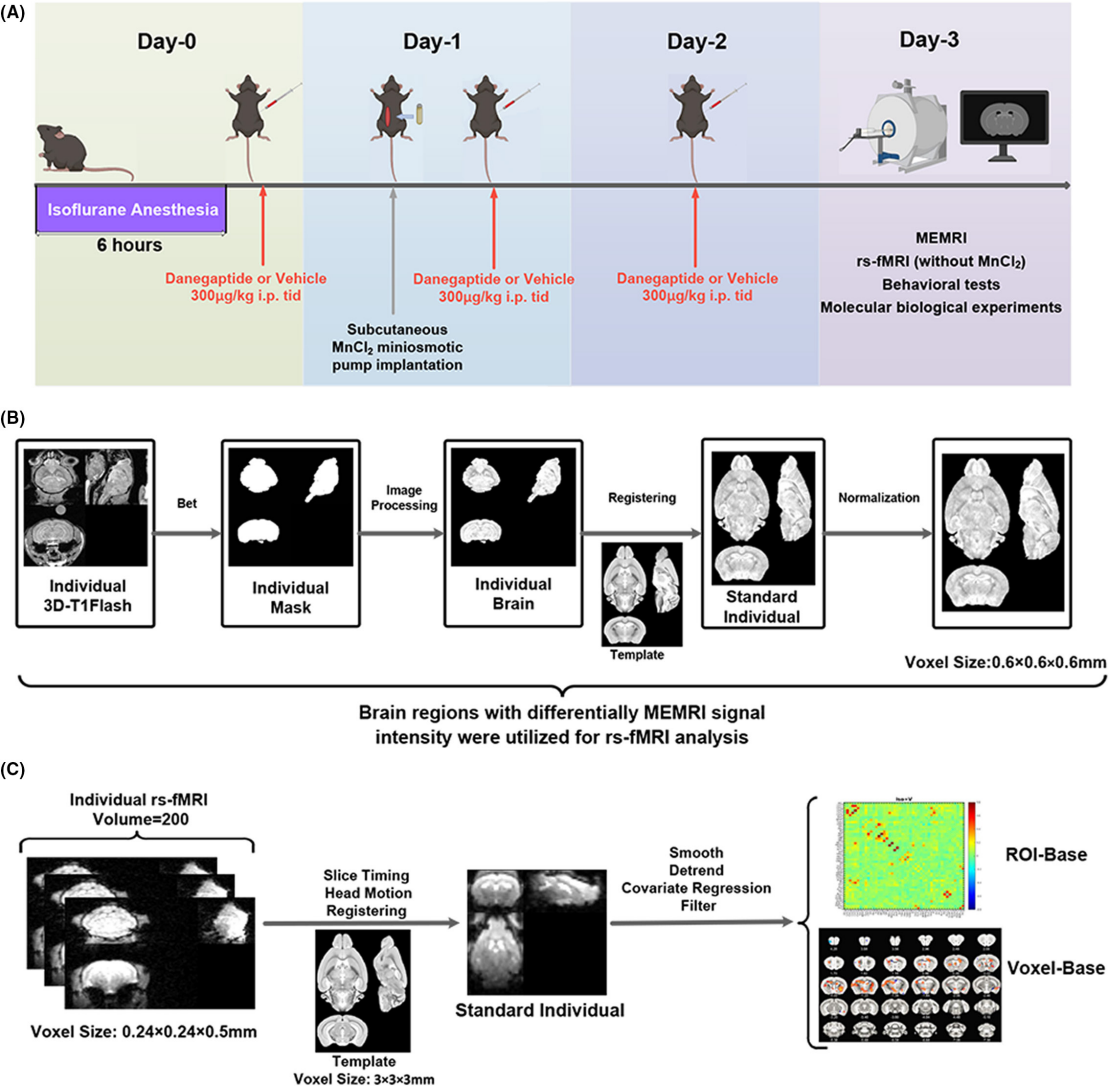
Temporal diagram depicting the groups, treatments, and procedures used in the magnetic resonance imaging (MRI) study. (A) Study flowchart for MRI. The animals were divided into three groups: Con + V, Iso + V, and Iso + D groups. Group Iso + D received long‐term isoflurane anesthesia and intermittent intraperitoneal injections of Danegaptide, group Iso + V received long‐term isoflurane anesthesia and intermittent intraperitoneal injections of vehicle (saline), and group Con + V received only intermittent injections of vehicle (saline), without long‐term isoflurane anesthesia. The pumping of MnCl_2_ is completed by osmotic pumping 24 h before the manganese‐enhanced magnetic resonance imaging (MEMRI). For rs‐fMRI (resting‐state functional magnetic resonance imaging), no pumping of MnCl_2_ was necessary. (B) MEMRI data processing and analysis. Brain regions with significant differences in MEMRI signal intensity were determined by voxel and ROI analysis. (C) rs‐fMRI data processing and analysis. All brain regions with differential MEMRI signal intensity were subjected to FC analysis. Furthermore, we used the bilateral hippocampus as seeds in the FC analysis of the whole brain. ROI, region of interest; FC, functional connectivity

Four‐month‐old male C57BL/6 mice were housed in the same room at constant temperature (22+ 1°C) and humidity (50+ 10%) and with a 12/12‐h light‐dark cycle (lights on between 8 am and 20 pm). The mice were allowed to acclimatize to the test environment for at least 1 week. A flow diagram of the animal experiments is shown in Figure 1A, where the mice were divided into three groups: mice were subjected to an intraperitoneal injection of vehicle (saline) alone (Con + V), mice were anesthetized with long‐term isoflurane and subjected to an intraperitoneal injection of vehicle (saline) (Iso + V), mice were anesthetized with long‐term isoflurane and subjected to an intraperitoneal injection of Danegaptide (a specific enhancer that promotes Cx43 to form gap junctions (gjs), GLPBIO, Cat: GC16618) (Iso + D). The Ethics Committee of the Affiliated Drum Tower Hospital of Nanjing University Medical School approved all animal studies (license number 2020AE01110).

### 2.2 Isoflurane anesthesia

Animal anesthesia was performed between 9 pm and 3 am according to a previous study.^4^ In brief, mice were placed in an anesthetizing box with 4.2% isoflurane in oxygen supplied until the righting reflex was lost and subsequently maintained with 1.2%–1.5% isoflurane throughout the procedure. Control mice underwent the same procedure, but without isoflurane.

The same anesthetic agent was applied to astrocytes at 37°C in a 5% CO_2_ incubator. The incubator was prefilled with 5% isoflurane, and the isoflurane concentration was adjusted to 1.5% after the astrocytes were placed inside. After 6 h, subsequent experiments were performed on astrocytes. Controls were treated in the same manner without isoflurane.

### 2.3 Behavioral test

In this study, Y‐maze and fear conditioning tests were used to evaluate the effect of long‐term isoflurane anesthesia on cognitive function in mice.

Fear conditioning tests were conducted to assess fear‐based learning and memory. In the training phase, mice were first placed in the training box for 3 min. Then, they were exposed to a high‐frequency sound (1000 Hz, 75 dB) for 30 s. During the final 2 s, a 0.8 mA (milliampere) foot shock was given. Another round of sound and foot shock stimuli was performed 60 s after the end of the previous round of sound and foot shock stimuli. After the second foot shock, mice were allowed to continue to explore the chamber for 60 s before returning to their home cages. For the contextual fear conditioning (CFC) test, mice were placed in the chamber for 5 min in the absence of sound and foot shock stimuli 24 h after the training, and the percentage of freezing time was recorded automatically by Tracking Master V3.0. For the tone associative fear conditioning (TFC) test, mice were exposed to the high‐frequency sound alone without foot shocks in a different context, and the percentage of time freezing during the 30 s presentation of sound was recorded.

Spatial working memory performance was evaluated by the Y‐maze test as described previously.^19^ Mice were placed in the center of the Y‐maze and allowed to freely explore the maze for 8 min. The trajectory of the mice was recorded by a video camera and transmitted to the EthoVision XT 11.5 for analysis. The percentage of spontaneous alternations was used as an index of hippocampus‐dependent working memory performance.^20^

### 2.4 Primary astrocyte culture

Primary astrocytes were prepared from neonatal mice as described previously.^21^ Briefly, the meninges were removed, and the cortical hemispheres were dissected into small pieces and then digested with 0.25% trypsin for 10 min at 37°C. Filtered cells were centrifuged and resuspended in Dulbecco's modified Eagle's medium (DMEM, KeyGEN BioTech, Cat:KGM12800‐500) with 10% fetal bovine serum (FBS). After being cultured for 5–7 days, astrocytes were purified by shaking the dishes to detach all other cells. The purity of the primary astrocytes was determined by glial fibrillary acidic protein (GFAP) immunofluorescence staining and flow cytometry analysis. Experiments were repeated with a different pool of breeding pairs.

### 2.5 GFAP immunofluorescence staining

Astrocytes on coverslips were fixed with 4% formaldehyde for 30 min. Anti‐GFAP antibody (1:100, Cell Signaling Technology, Cat: 3670S) was incubated overnight at 4°C, and anti‐mouse Alexa Fluor® 488 (1:1000, Abcam, Cat: ab150113) was incubated for 2 h at room temperature. Images were captured by the Thunder Imaging System (Leica, Germany). The astrocytes were counted with ImageJ software version 1.53c (NIH, USA).

### 2.6 Flow cytometry

Astrocyte purity was determined by flow cytometry analysis for GFAP^+^ cells as previously described in the literature.^21^ The antibody used for flow sorting was Alexa Fluor® 488 Mouse anti‐GFAP (1:50, cat: 561449, BD Pharmingen, USA). Data were acquired on the BD Accuri™C6 Flow Cytometer (BD Biosciences, USA) and analyzed with FlowJo software 10.0 (TreeStar Inc., Ashland, OR, USA).

### 2.7 Preparation of soluble and insoluble fractions of Cx43

Hippocampal tissues and primary astrocytes were collected and the total protein was extracted using RIPA Lysis Buffer (P0013B, Beyotime, China) on ice. The extraction method of Cx43 forming GJ configuration is based on the ability of GJs‐Cx43 to be insoluble in 1% Triton X‐100.^11^, ^22^ In brief, hippocampal tissues and primary astrocytes were collected and homogenized on ice with a homogenizer in immunoprecipitation buffer containing 1% Triton X‐100, incubated on ice for 30 min, and centrifuged at 12,000 rpm (revolutions per minute) for 30 min at 4°C and the supernatant (soluble fractions of Cx43) was collected. The precipitates were resuspended in immunoprecipitation buffer containing 1% Triton X‐100 and 4 M urea. Subsequently, the homogenate was centrifuged and supernatants (insoluble fractions of Cx43) were collected. The increase of insoluble fraction indicates that Cx43 forming GJs is increased.^11^

### 2.8 Western blotting

Equal amounts of protein were separated by 10% SDS‐polyacrylamide gel electrophoresis (PAGE) and then transferred to polyvinylidene difluoride (PVDF) membranes. The membrane was blocked for 90 min in blocking buffer (5% skim milk and 0.1% Tween 20 in phosphate‐buffered saline (PBS)). Primary antibodies were incubated overnight at 4°C, and secondary antibodies were incubated for 2 h at room temperature. The primary antibodies used were: anti‐Connexin43 (1:16000, Sigma, Cat: C6219), anti‐IL‐1β (0.2 μg/ml, Abcam, Cat: ab9722), anti‐IL‐6 (1:1000, Abcam, Cat: ab6672), and anti‐β‐tubulin (1:7500, Beyotime, Cat: AF1216) or anti‐β‐actin (1:10000, Proteintech, Cat: 66009‐1‐1g). The signal in the PVDF membranes was detected using a chemiluminescent solution (Thermo Fisher, Cat: 34580) and scanned using a Tanon‐4600 Chemiluminescent Imaging System (Tanon, China). The quantification of Western blot bands was carried out using ImageJ software version 1.53c (NIH, USA) normalized by β‐tubulin or β‐actin.

### 2.9 Reactive oxygen species (ROS) detection assay

The ROS activity was detected by the ROS detection probe 2′,7′‐dichlorofluorescein diacetate (DCFH‐DA) (Beyotime, Cat: S0033S) combined with flow cytometry analysis. ROS can oxidize nonfluorescent DCFH to DCF with green fluorescence, so that DCF fluorescence intensity can indicate intracellular ROS levels. Freshly isolated hippocampal cells or primary astrocytes were loaded with DCFH‐DA as per the manufacturer's instructions, and positive signal cells were retrieved by the BD Accuri^TM^C6 Flow Cytometer (BD Biosciences, USA).

### 2.10 Measurement of superoxide dismutase (SOD) and malondialdehyde (MDA)

Malondialdehyde (MDA) levels in hippocampal tissue and primary astrocytes were measured by the lipid peroxidation MDA assay kit (Nanjing Jiancheng, Cat: A003‐1, China) following the manufacturers' protocol. SOD activity in the hippocampal tissue and primary astrocytes was assessed using a SOD Assay Kit‐WST (Nanjing Jiancheng, Cat: A001‐3, China) according to the manufacturer's instructions.

### 2.11 MnCl_2_ administration and MEMRI data acquisition

MnCl_2_ (M813486, Macklin, China) was dissolved at a concentration of 100 mM in BICINE (N,N‐Bis(2‐hydroxyethyl)glycine) (B3876, Sigma, USA) and the pH was adjusted to 7.4 using NaOH. The prepared solution was stored at 4°C and diluted with BICINE (400 Mm) before each experiment. An Alzet osmotic minipump (Model 2001D, Durect Corporation, Cupertino, CA) filled with MnCl_2_ was subcutaneously implanted in the right side of the abdomen in mice. A constant flow rate of 8 μl per hour was maintained for each minipump until it had delivered 200 μl of MnCl_2_ solution by continuous infusion. The concentration of MnCl_2_ solution in the minipump was adjusted according to the body weight of each mouse to ensure that the final total amount of MnCl_2_ injected per mouse was 50 mg/kg. All mice completed MnCl_2_ working solution pumping 24 h before MEMRI scanning. During the pump implantation and MnCl_2_ infusion, there was no infection or weight loss in any group (Figure S1).

The MEMRI data were acquired using a 9.4 T MRI scanner (BioSpec 94/20 USR, Bruker, Germany), with an 86‐mm‐diameter volume coil for radiofrequency (RF) pulse transmission and a surface coil for signal detection. Mice were anesthetized with medetomidine and isoflurane according to reference^23^ and placed in the head‐fixed setup at the center of the mouse coil. The MEMRI images were obtained using a three‐dimensional T1WI fast low angle shot (3D T1WI‐FLASH) sequence, field of view 15.6 mm × 15.6 mm × 19.2 mm, matrix 156 × 156 × 128, voxel size 0.100 mm × 0.100 mm × 0.150 mm, repetition time (TR) 20 ms, echo time (TE) 4.1 ms, excitation flip angle 15°, and 3 averages. The total acquisition time for each MEMRI scan was 22 min and 24 s. The spontaneous breathing rate was monitored by a respiratory pneumatic sensor placed underneath the abdomen of the mice and maintained within a target range of 85–110 breaths per minute by slight adjustments to isoflurane concentration. Mouse body temperature was monitored using a rectal thermometer and was kept constant at 37 ± 1.0°C with a warm water circulation system (HAAKE SC100, Thermo Scientific, USA). Arterial blood from the abdominal aorta was collected for blood gas analysis immediately after the MEMRI scan to exclude nonphysiological conditions.

### 2.12 MEMRI data processing

Our study applied two different statistical approaches for MEMRI analyses: voxel‐wise comparisons and region of interest (ROI)–based comparisons. Figure 1C gives an overview of our data processing flow. First, scalp, skull, dura, and other nonbrain tissues were removed from the T1WI‐FLASH images by automated and manual skull stripping. Next, volume‐based spatial normalization to one standard space was performed with open‐source Advanced Normalization Tools (http://picsl.upenn.edu/software/ants/). For each animal, the voxel‐wise MEMRI signal intensity was normalized to the mean MEMRI signal intensity of the whole brain. Voxel‐wise comparisons at the group level were performed with two‐tailed independent samples t‐test and the statistical significance was set at voxel‐level *p* < 0.01, cluster‐level *p* < 0.05, Gaussian Random Field (GRF) corrected. For ROI analyses, a mice brain atlas (TMBTA (Turone Mouse Brain Atlas and Template)) was used to guide ROI placement,^24^ and ROIs were extracted from the voxel‐wise analysis results using an in‐house script. The ROI masks were subsequently superimposed onto the normalized images of each animal to determine the average signal intensity of Mn^2+^. ROI comparisons at the group level were performed with a two‐tailed independent samples t‐test and false discovery rate (FDR) correction. The volume in the ROI was calculated from the number of voxels within the ROI. All activation maps were overlaid on brain template images for more accurate visualization.

### 2.13 rs‐fMRI data acquisition

Magnetic resonance imaging (MRI) acquisition was performed as described above. To ensure the correct positioning of the mouse head, a 3‐plane pilot scan was acquired using a fast location image, followed by a FieldMap with a consecutive local shim to optimize the magnetic field homogeneity and image quality. Subsequently, whole‐brain rs‐fMRI scans were acquired using an Echo Planar Imaging (EPI) sequence. Each scan consisted of 200 volumes obtained by the following parameters: TR = 1500 ms, TE = 7.9 ms, flip angle = 15°, spatial resolution = 0.24 mm × 0.24 mm, field of view (FOV) = 30 mm × 12 mm, slice thickness = 0.5 mm, and 35 slices in total, and the duration of the rs‐fMRI acquisition time was 5 min. The following parameters were used when acquiring an additional T2‐weighted anatomical scan in the same geometry as the rs‐fMRI scan: TR = 4000 ms, effective TE = 33 ms, rapid acquisition with relaxation enhancement (RARE) factor = 8, spatial resolution = 0.07 mm × 0.07 mm × 0.5 mm, and matrix = 256 × 256.

### 2.14 rs‐fMRI data processing

The rs‐fMRI data were preprocessed before performing an FC analysis which included voxel amplification (10 times), deletion of the first 10 points, slice timing, head movement correction, spatial standardization, smoothing, detrending, filtering, and covariate regression. Brain FC was assessed using a seed‐based approach by DPABI (Data Processing and Analysis for Brain).^25^ Voxel‐wise comparisons at the group level were performed with two‐tailed independent samples t‐test and the statistical significance was set at voxel‐level *p* < 0.01, cluster‐level *p* < 0.05, GRF corrected. ROI comparisons at the group level were performed with two‐tailed independent samples *t*‐test, FDR corrected. On the brain template images, color‐encoded percentage signal changes were superimposed to demonstrate fMRI activation.

### 2.15 Arterial blood gas analysis

After the MEMRI or rs‐fMRI examination, 0.2 ml abdominal aorta blood was immediately collected in heparinized syringe. The i‐STAT portable blood‐gas analyzer (Abbott Park, IL, USA) and the i‐STAT CG8+ cartridge (Abbott Park, IL, USA) were used to measure arterial blood gas.

### 2.16 Statistical analysis

SPSS 21.0 (SPSS, IBM, USA) was used for statistical analysis. Data are expressed as means ± SD. The normality of values was tested with the Kolmogorov‐Smirnov test. Levene's test was applied to test the homogeneity of variance. Two‐tailed independent samples *t*‐test was employed to compare the data between two groups. Multivariate data were analyzed with one‐way analysis of variance (ANOVA) followed by a Bonferroni test when variances were homogeneous or by the Dunnett T3 test when variances were not homogeneous. The Mann‐Whitney *U* test was used if the data did not meet a normal distribution. If not otherwise indicated, *p* < 0.05 was considered the level of statistical significance.

## 3 RESULTS

### 3.1 Increasing GJs‐Cx43 coupling attenuated long‐term isoflurane anesthesia–induced cognitive impairment

**FIGURE 2:**
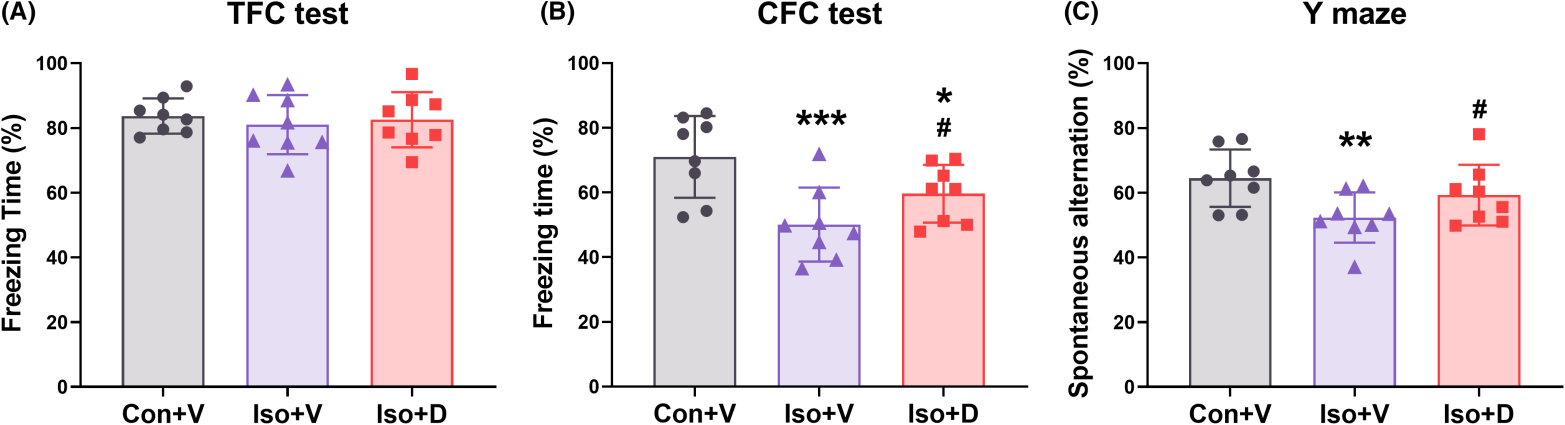
Enhanced gap junctions Connexin43 (GJs‐Cx43) coupling can alleviate cognitive dysfunction induced by long‐term isoflurane anesthesia. Cognitive functions are impaired in mice after long‐term isoflurane anesthesia and treatment with Danegaptide (GJs‐Cx43 enhancer) significantly improved the contextual fear memory (A) and working memory (C) without affecting cued fear memory (B). Data are presented as the mean ± SD. *p < 0.05, **p < 0.01, ***p < 0.001 compared to the Con + V group, ^#^ *p* < 0.05, ^##^ *p* < 0.01, ^###^ *p* < 0.001 compared to the Iso + V group.

The mice were given three intraperitoneal Danegaptide injections (300 μg/kg) per day for three consecutive days according to the previous study.^11^ There was no significant difference among the three groups in the freezing time during the training phase of the fear conditioning tests (Figure S2A, *F*[2,21] = 0.386, *p* = 0.682). The freezing time in the tone associative fear condition (TFC) test was not significantly different among the three groups (Figure 2A, *F*[2,21] = 0.332, *p* = 0.719). The freezing time was significantly reduced in the Iso + D group and Iso + V group compared to the Con + V group in the CFC test, whereas Danegaptide administration significantly increased the freezing time in Iso mice (Figure 2B, *F*[2,21] = 11.805, *p* < 0.001). In the Y‐maze test, there was no significant difference in movement velocity or visited arms rate among the different groups (Figure S2B,C). Compared with the Con + V group, the mice in the Iso + V group displayed fewer spontaneous alterations, which were reversed by the Danegaptide treatment (Figure 2C, *F*[2,21] = 6.162, *p* = 0.004). The aforementioned data indicated that increasing GJs‐Cx43 coupling could alleviate the impairment of cognition driven by long‐term isoflurane anesthesia.

### 3.2 Mapping the activity of the whole brain in mice using MEMRI

**FIGURE 3:**
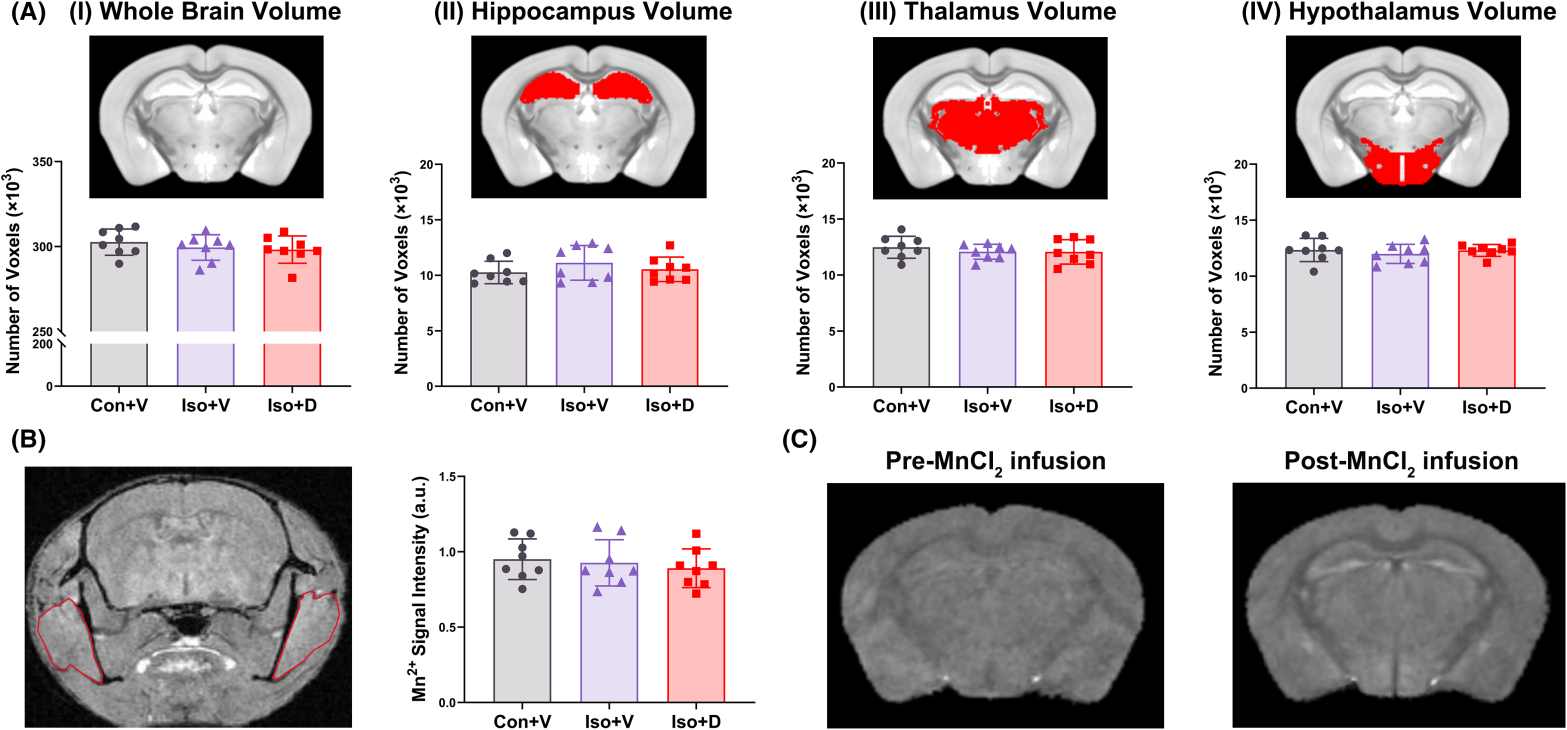
Comparison of brain volume and MnCl_2_ absorption rate in mice. (A) Voxel‐based morphometry was used to assess voxel‐wise differences in whole‐brain volume (I), hippocampal volume (II), thalamus volume (III), and hypothalamic volume (IV). Each image has an inset attached to it showing specifically the regions of interest (ROI) (red shading). All the above region volumes were not statistically different among the three groups (*n* = 8/group). (B) The absorption rate of MnCl_2_ in the three groups did not show any significant difference 24 h after the end of MnCl_2_ infusion (*n* = 8/group). Left panel: A schematic illustration representing the temporalis muscle (red circle) that we used. Right panel: Quantitative analysis of manganese‐enhanced magnetic resonance imaging (MEMRI) signal intensity in the bilateral temporalis muscle. (C) Representative registered T1‐weighted (T1WI) fast low angle shot sequence (T1W1‐FLASH) images obtained pre‐ and post‐MnCl_2_ infusion. Data are presented as the mean ± SD

The physiology of anesthetized mice was carefully monitored and controlled throughout the MEMRI, and blood gas analyses were performed after the MEMRI scan. No statistical differences were observed among the three groups (Con + V, Iso + V, and Iso + D) regarding physiological parameters (Table 1). There were no significant differences in total brain, thalamic, and hippocampal volumes among the three groups (Figure 3A). Figure 3B plots the raw MEMRI signal intensities in the bilateral temporal region, and there was no difference among the three groups. These analyses were performed to confirm that the availability of Mn^2+^ was not different between the groups.^26^ In addition, to show the enhancement effect of Mn^2+^ on brain signals more intuitively, the registered T1WI‐FLASH images pre‐ and post‐MnCl_2_ infusion are shown in Figure 3C.

**TABLE 1:** Physiologic parameters obtained from mice that underwent MEMRI (manganese‐enhanced magnetic resonance imaging)

	Con + V	Iso + V	Iso + D	F	*p*
PH	7.39 ± 0.04	7.38 ± 0.04	7.38 ± 0.03	0.041	0.960
PCO_2_ (mmHg)	38.79 ± 3.18	39.43 ± 2.67	38.75 ± 1.38	0.481	0.622
PO_2_ (mmHg)	158.75 ± 24.09	158.88 ± 18.49	154.88 ± 23.00	0.198	0.821
HCO_3_^−^ (mmol/L)	20.49 ± 1.94	21.03 ± 1.79	20.63 ± 1.00	0.490	0.616
SO2 (%)	98.50 ± 0.53	98.36 ± 0.50	98.25 ± 0.44	0.915	0.408
Na^+^ (mmol/L)	145.88 ± 3.31	145.75 ± 2.91	146.63 ± 2.45	0.555	0.578
K^+^ (mmol/L)	3.16 ± 0.39	3.29 ± 0.37	3.18 ± 0.25	0.711	0.496
Ca^2+^(mmol/L)	1.30 ± 0.06	1.32 ± 0.04	1.30 ± 0.06	1.070	0.352
Glu (mg/dl)	348.75 ± 54.19	367.38 ± 46.17	336.63 ± 65.70	1.345	0.271
Hct (%)	43.00 ± 2.00	41.88 ± 2.28	41.50 ± 1.98	1.552	0.223
Hb (g/dl)	14.38 ± 0.82	14.58 ± 0.70	14.69 ± 0.60	0.662	0.521

*Note*: Data are presented as the mean ± SD (*n* = 8/group).

**FIGURE 4:**
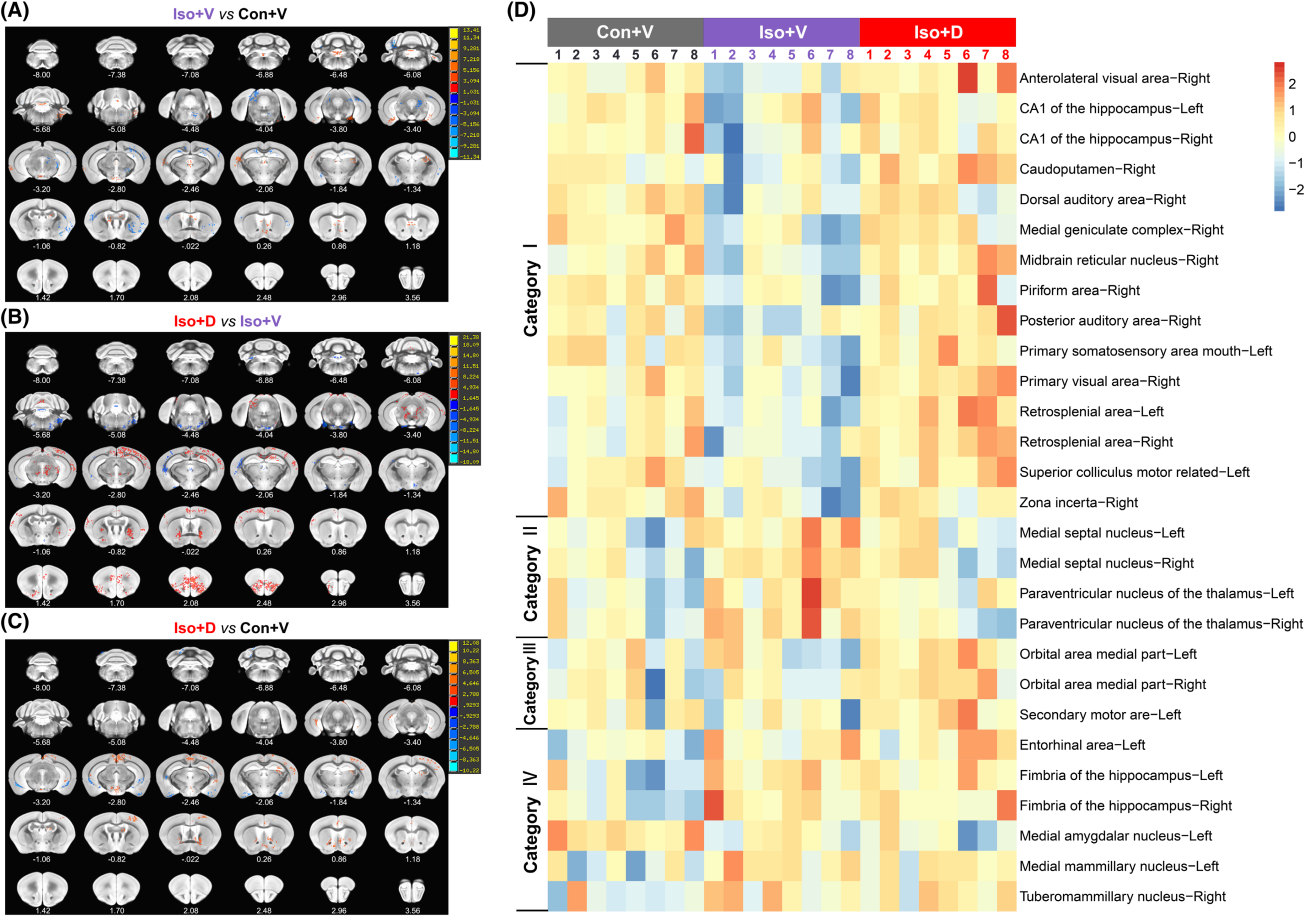
Voxel‐wise whole‐brain and ROI‐based classification analyses for manganese‐enhanced magnetic resonance imaging (MEMRI) under different interventions. (A) Whole‐brain maps of significant differences in MEMRI signal intensity between the Iso + V group and Con + V group. Voxels colored blue indicate brain areas in which the Iso + V group has reduced MEMRI signal intensity compared with the Con + V group, and voxels colored red indicate brain areas in which the Iso + V group has abnormally increased MEMRI signal intensity (*n* = 8/group, two‐tailed independent samples t‐test, voxel‐level *p* < 0.01, cluster‐level *p* < 0.05, GRF correction). (B) Whole‐brain maps of significant differences in MEMRI signal intensity between the Iso + D group and Iso + V group. The blue voxels indicate brain areas in which the Iso + D group has reduced MEMRI signal intensity compared with the Iso + V group, and the red voxels indicate brain areas in which the Iso + D group has abnormally increased MEMRI signal intensity(*n* = 8/group, two‐tailed independent samples *t*‐test, voxel‐level *p* < 0.01, cluster‐level *p* < 0.05, GRF correction). (C) Whole‐brain maps of significant differences in MEMRI signal intensity between the Iso + D group and Con + V group. The blue voxels indicate brain areas in which the Iso + D group has reduced MEMRI signal intensity compared with the Con + V group, and the red voxels indicate brain areas in which the Iso + D group has abnormally increased MEMRI signal intensity (*n* = 8/group, two‐tailed independent samples *t*‐test, voxel‐level *p* < 0.01, cluster‐level *p* < 0.05, GRF correction). The numbers in the corner of each image represent the anatomical distance from the bregma. GRF: Gaussian random field. (D) All 28 brain regions listed in the right ordinate were statistically different (*p* < 0.05, FDR correction). The color bars on the up abscissa represent the grouping of samples (*n* = 8/group), and the color numbers represent different samples. The left ordinate represents the clustering result of the brain regions. The color of the cell indicates the relative MEMRI signal intensity: the redder the color, the higher the MEMRI signal intensity; the bluer the color, the lower the MEMRI signal intensity. FDR: false discovery rate, ROI: region of interest.

The results of the voxel‐based whole‐brain analyses for MEMRI data are displayed in Figure 4A–C. The alterations in whole‐brain MEMRI signal intensity induced by long‐term isoflurane anesthesia are brain region–specific, and the changing trend of MEMRI signal intensity is not completely consistent in different brain regions. Overall, the tendency to vary the MEMRI signal intensity in Iso + V versus Con + V was the opposite of that in the Iso + D versus Iso + V (Figure 4A,B). Nevertheless, brain regions show significant activation differences in whole‐brain voxel‐wise comparisons between Iso + D and Con + V (Figure 4C).

To better understand the role of individual brain areas in our models, we then applied ROI‐based comparisons to independently validate the voxel‐wise comparisons. Based on the results of voxel analysis, we extracted 50 brain regions for ROI analysis with reference to the mice brain atlas, and finally found statistical differences in 28 brain regions. As expected from the results shown in Figure 4D, four categories of the 28 brain regions were classified according to the effect of increased GJs‐Cx43 coupling on the MEMRI signal intensity in the mouse brain after long‐term isoflurane exposure. Category I: Reversed activation, which means long‐term isoflurane anesthesia–induced reduction of MEMRI signal intensity is reversed by GJs‐Cx43 coupling. This category includes the anterolateral visual area‐right, CA1 of the hippocampus‐bilateral, caudoputamen‐right, dorsal auditory area‐right, medial geniculate complex‐right, midbrain reticular nucleus‐right, piriform area‐right, posterior auditory area‐right, primary somatosensory area mouth‐left, primary visual area‐right, retrosplenial area‐bilateral, superior colliculus motor related‐left, and zona incerta‐right. Category II: Reversed inhibition, which means that long‐term isoflurane anesthesia causes MEMRI signal intensity but is reversed by GJs‐Cx43 coupling. This category includes the medial septal nucleus‐bilateral and paraventricular nucleus of the thalamus‐bilateral. Category III: Intensified activation, which means that MEMRI signal intensity showed no statistical differences between group Con + V and group Iso + V, but increases in group Iso + D, including the orbital area medial part‐bilateral and secondary motor area‐left. Category IV: No‐effect, which means that GJs‐Cx43 coupling had no effect on the change of MEMRI signal intensity caused by long‐term isoflurane anesthesia, including the entorhinal area‐left, fimbria of the hippocampus‐bilateral, medial amygdalar nucleus‐left, medial mammillary nucleus‐left, and tuberomammillary nucleus‐right. These results suggest that isoflurane‐induced cognitive dysfunction is a complex network, rather than a region‐based disease, and GJs‐Cx43 decoupling also involves engaging multiple brain regions.

### 3.3 Reconfiguring the GJs‐Cx43–mediated astrocytic network influences FC between the brain regions that have significant differences in MEMRI

**FIGURE 5:**
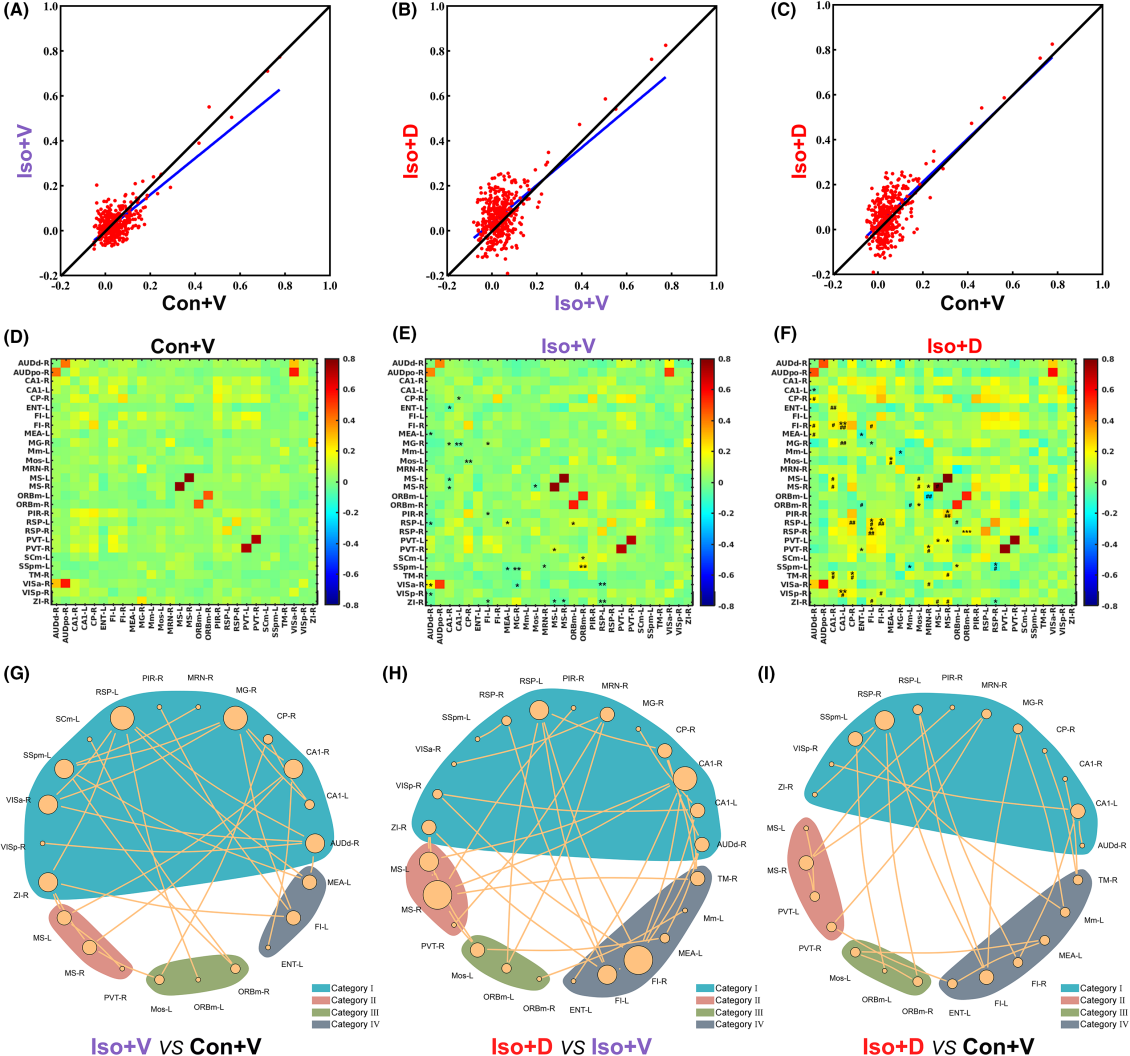
FC analysis was performed in brain regions with different manganese‐enhanced magnetic resonance imaging (MEMRI) signal intensities. (A) Comparison of each ROI‐ROI correlation coefficient between Iso + V and Con + V groups. The overall changing trend of the FCs presented a general decrease after long‐term isoflurane anesthesia. (B) Comparison of each ROI‐ROI correlation coefficient between the Iso + D and Iso + V groups. The FCs showed a general trend of increasing after the Danegaptide interventions. (C) Comparison of each ROI‐ROI correlation coefficient between the Iso + D and Con + V groups. The obtained FCs do not show an obvious trend with the distance y = x. Averaged connectivity matrices in Con + V(D), Iso + V(E), and Iso + D(F) groups, the color indicates the relative FC strength, and the color red represents excitatory connections, the color blue represents inhibitory connections (*n* = 8/group; *p < 0.05, **p < 0.01, ***p < 0.001 compared to the Con + V group, ^#^ *p* < 0.05, ^##^ *p* < 0.01, ^###^ *p* < 0.001 compared to the Iso + V group; two‐tailed independent samples *t*‐test, FDR uncorrected). (G) The network topographs of FCs with statistically significant differences between Iso + V and Con + V groups. (H) The network topographs of FCs with statistically significant differences between Iso + D and Iso + V groups. (I) The network topographs of FCs with statistically significant differences between Iso + D and Con + V groups; the yellow bubble symbols represent the ROI, and the lines the FC. The larger the yellow bubble, the more connections are included. ROIs are clustered based on their changes in MEMRI signal intensity. See Table 3 for the full names of abbreviated ROIs. FC: functional connectivity, FDR: false discovery rate, ROI: region of interest

We used the brain regions with differential MEMRI signal intensity as ROI, and FC was analyzed between these brain regions (Table 2). No significant differences were observed in physiological parameters among the different groups during the rs‐fMRI scanning sessions (Table 3). As shown in Figure 5A, 154 connections were above the central line (y = x) and 224 connections were below it, which indicate that the connectivity strength was significantly decreased in the Iso + V group. In Figure 5B, 240 connections were above the central line (y = x) and 138 connections below it, which means that Danegaptide elevated FC between brain regions compared to the Iso + V group. However, compared with the Con + V group (Figure 5C), the Iso + D group also showed increased FC (225 connections above the central line and 153 connections below it). This indicates that the long‐term isoflurane exposure appears to cause FC strength to fall, whereas promoting GJs‐Cx43 coupling enhanced FC strength in mice after a long‐term isoflurane anesthesia. The strength of FC between two different brain regions is illustrated by the color of every cell in the matrix (Figure 5D–F). Twenty‐eight connections among 22 brain regions were significantly different between the Iso + V and Con + V groups (Figure 5D,G, all *p* < 0.05, FDR corrected). Compared with the Iso + V group, promoting GJs‐Cx43 coupling resulted in changes in 33 connections among 22 brain regions(Figure 5E,H, all *p* < 0.05, FDR corrected). We also compared the changes in FCs between the Iso + D and Con + V groups, and found that there were significant differences in 24 connections among the 25 brain regions(Figure 5F,I, all *p* < 0.05, FDR corrected). In terms of the differentially altered FCs, promoting GJs‐Cx43 coupling can specifically reverse the decrease in FC between the MEA‐L & AUDd‐R, CA1‐R & ENT‐L, CA1‐R & MS‐L, CA1‐R & MS‐R, CA1‐L & MG‐R, MS‐R & Mos‐L, ZI‐R & FI‐L, ZI‐R & MS‐L, and ZI‐R & MS‐R caused by long‐term isoflurane anesthesia, and the increase in FC between the RSP‐L & ORBm‐L (see Table 3 for explanations of these abbreviations). The results described above suggest that GJs‐Cx43 coupling can partially reverse the abnormal FCs induced by long‐term isoflurane anesthesia, and with compensatory changes in other FCs.

**TABLE 2:** Physiologic parameters obtained from mice that underwent resting‐state functional magnetic resonance imaging (rs‐fMRI)

	Con + V	Iso + V	Iso + D	*F*	*p*
PH	7.40 ± 0.03	7.38 ± 0.02	7.39 ± 0.03	0.514	0.601
PCO_2_ (mmHg)	37.69 ± 3.34	38.26 ± 3.04	39.16 ± 2.61	0.968	0.388
PO_2_ (mmHg)	157.63 ± 24.66	155.50 ± 9.81	154.37 ± 14.11	0.141	0.869
HCO_3_^−^ (mmol/L)	20.69 ± 1.80	20.93 ± 1.51	20.61 ± 1.35	0.216	0.806
SO2 (%)	98.75 ± 0.46	98.63 ± 0.50	98.50 ± 0.51	0.833	0.441
Na^+^ (mmol/L)	146.88 ± 2.42	145.88 ± 3.68	146.50 ± 2.75	0.343	0.711
K^+^ (mmol/L)	3.18 ± 0.41	3.36 ± 0.32	3.24 ± 0.24	1.302	0.282
Ca^2+^(mmol/L)	1.31 ± 0.07	1.33 ± 0.04	1.29 ± 0.07	2.030	0.143
Glu (mg/dl)	333.50 ± 65.29	339.25 ± 48.73	332.63 ± 59.00	0.068	0.935
Hct (%)	42.88 ± 2.30	42.13 ± 2.60	42.13 ± 1.94	0.375	0.690
Hb (g/dl)	14.28 ± 0.75	14.71 ± 0.69	14.41 ± 0.61	1.493	0.236

*Note*: Data are presented as the mean ± SD (*n* = 8/group).

**TABLE 3:** List of regions of interest (ROIs) included in functional connectivity (FC) analysis

ROI Name	Abbreviation	ROI Name	Abbreviation
Dorsal auditory area‐Right	AUDd‐R	Medial septal nucleus‐Right	MS‐R
Posterior auditory area‐Right	AUDpo‐R	Orbital area medial part‐Left	ORBm‐L
CA1 of the hippocampus‐Right	CA1‐R	Orbital area medial part‐Right	ORBm‐R
CA1 of the hippocampus‐Left	CA1‐L	Piriform area‐Right	PIR‐R
Caudoputamen‐Right	CP‐R	Retrosplenial area‐Left	RSP‐L
Entorhinal area‐Left	ENT‐L	Retrosplenial area‐Right	RSP‐R
Fimbria of the hippocampus‐Right	FI‐R	Paraventricular nucleus of the thalamus‐Left	PVT‐L
Fimbria of the hippocampus‐Left	FI‐L	Paraventricular nucleus of the thalamus‐Right	PVT‐R
Medial amygdalar nucleus‐Left	MEA‐L	Superior colliculus motor related‐Left	SCm‐L
Medial geniculate complex‐Right	MG‐R	Primary somatosensory area mouth‐Left	SSpm‐L
Medial mammillary nucleus‐Left	Mm‐L	Tuberomammillary nucleus‐Right	TM‐R
Secondary motor area‐Left	Mos‐L	Anterolateral visual‐area‐Right	VISa‐R
Midbrain reticular nucleus‐Right	MRN‐R	Primary visual area‐Right	VISp‐R
Medial septal nucleus‐Left	MS‐L	Zona incerta‐Right	ZI‐R

### 3.4 Whole‐brain resting‐state FC maps with the seed region in the hippocampus

**FIGURE 6:**
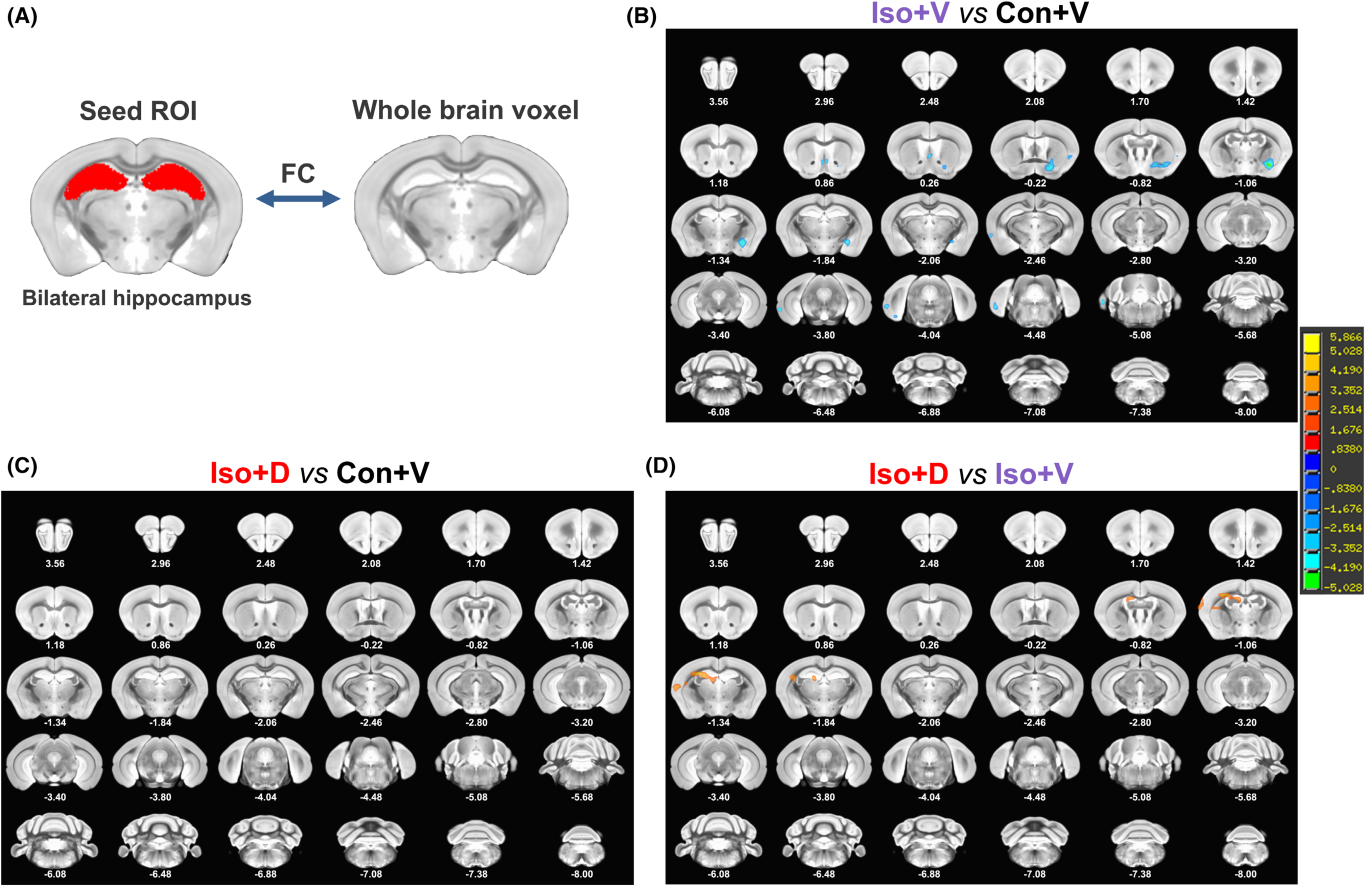
Whole‐brain FC analysis with the hippocampus as a seed region. (A) Schematic drawing of the ROI (red shading), whole‐brain connections were assessed by seed‐based functional analysis with the bilateral hippocampus as the seed region. (B) Significant increase in FC (hot color) and decrease in FC (blue color) in the Iso + V group compared with Con + V group (*n* = 8/group, two‐tailed independent samples *t*‐test, voxel‐level *p* < 0.01, cluster‐level *p* < 0.05, GRF correction), the brain regions with the most significantly reduced FC links were the nucleus accumbens‐right (ACB‐R), caudoputamen‐right (CP‐R), gustatory areas‐right (GU‐R), medial septal nucleus‐bilatum (MS), entorhinal area‐left (ENT‐L), substantia innominate‐right (SI‐R), fundus of striatum‐right (FS‐R), central amygdalar nucleus medial part‐right (CEAm‐R), and medial amygdalar nucleus‐right (MEA‐R). (C) There were no significant differences in the FC of the other brain regions with the bilateral hippocampus between the Iso + D and Con + V groups (*n* = 8/group, two‐tailed independent samples *t*‐test, voxel‐level *p* < 0.01, cluster‐level *p* < 0.05, GRF correction). (D) Significant increase in FC (hot color) and decrease in FC (blue color) in the Iso + D group compared with Iso + V group (*n* = 8/group, two‐tailed independent samples *t*‐test, voxel‐level *p* < 0.01, cluster‐level *p* < 0.05, GRF correction), bilateral hippocampus functional connectivity with the following brain regions were increased: caudoputamen‐left (CP‐L), supplemental somatosensory area‐left (SSs‐L), CA3 of hippocampus‐left (CA3‐L), and dentate gyrus‐left (DG‐L). FC, functional connectivity; GRF, Gaussian random field; ROI, region of interest

The hippocampus is a critical region for memory and learning and is involved in the pathophysiology of PND.^6^, ^7^, ^27^, ^28^ In addition, we found that the MEMRI signal intensity in the hippocampus decreased significantly after long‐term isoflurane anesthesia. Therefore, a seed‐based analysis was performed with the hippocampus as the seed region (Figure 6A). As showcased in Figure 6B, compared to the Con + V group, significant FC decreases in some areas were observed in the Iso + V group, and these differences mainly occurred in the nucleus accumbens‐right (ACB‐R), caudoputamen‐right (CP‐R), gustatory areas‐right (GU‐R), bilateral MS, entorhinal area‐left (ENT‐L), substantia innominate‐right (SI‐R), fundus of striatum‐right (FS‐R), central amygdalar nucleus medial part‐right (CEAm‐R), and medial amygdalar nucleus‐right (MEA‐R). These changes caused by long‐term isoflurane anesthesia were reversed by Danegaptide (Figure 6C). Furthermore, compared to the Iso + V group, we found that Danegaptide especially increased the FC between the hippocampus and the following brain regions (Figure 6D): caudoputamen‐left (CP‐L), supplemental somatosensory area‐left (SSs‐L), CA3 of hippocampus‐left (CA3‐L), and dentate gyrus‐left (DG‐L). The results described above show that long‐term isoflurane anesthesia can cause abnormal FCs from the hippocampus to other brain regions, and these abnormal changes can be reversed by reconstructing the GJs‐Cx43–mediated astrocytic network. Undoubtedly, these significantly different neural circuits have important research significance for the next stage.

### 3.5 Increasing GJs‐Cx43 coupling attenuated long‐term isoflurane‐induced oxidative stress and the inflammatory response in vitro and in vivo

**FIGURE 7:**
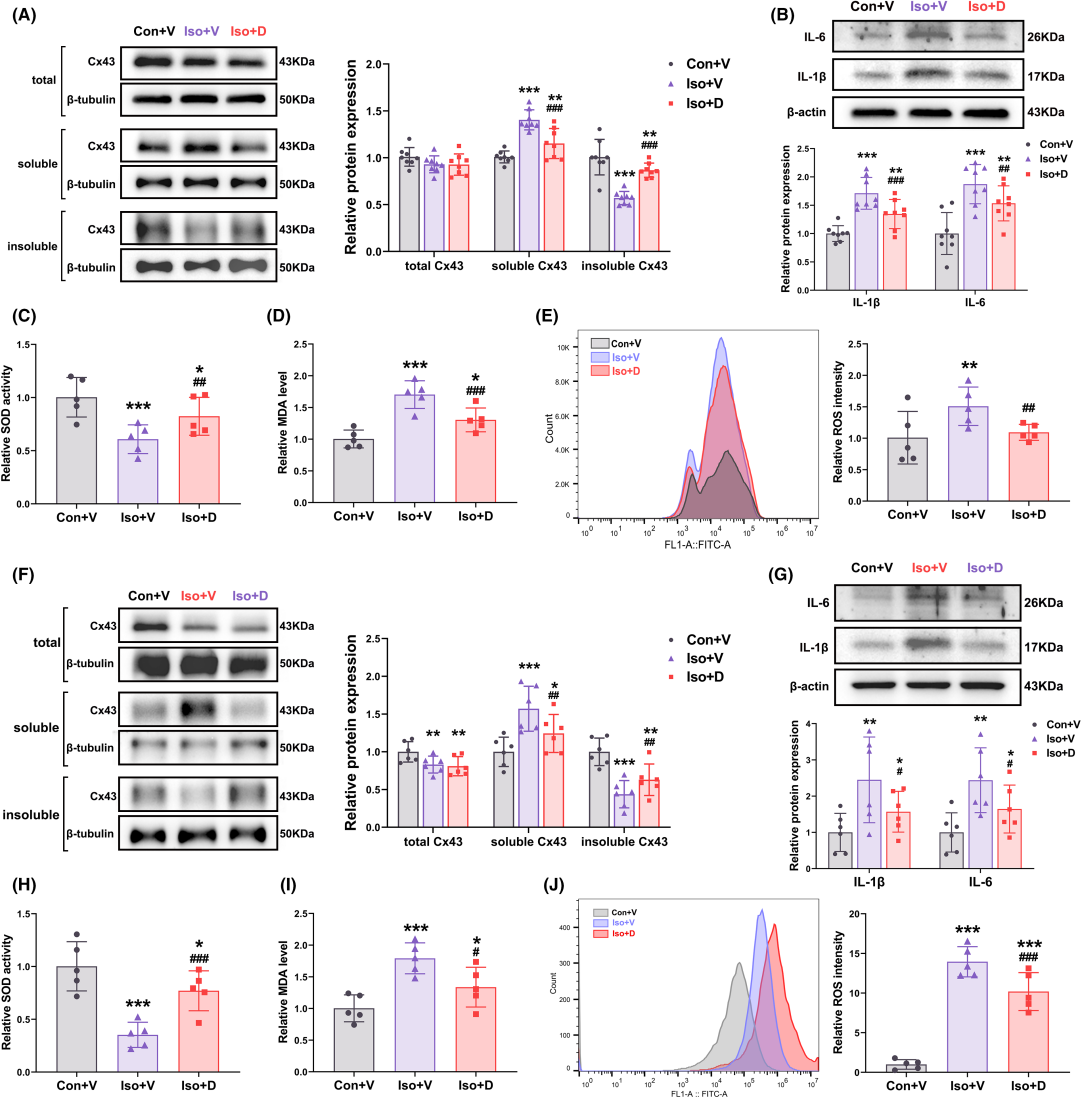
Increasing gap junctions Connexin43 (GJs‐Cx43) coupling attenuates oxidative stress and neuroinflammation in hippocampus and primary cultured astrocytes. (A) Representative bands of different Cx43 configurations in the hippocampus (left panel) and their quantification (right panel) (*n* = 6/group). (B) Representative bands of IL‐1β and IL‐6 in the hippocampus (upper panel) and their quantification (lower panel) (*n* = 6/group). (C) Superoxide dismutase (SOD) activity in all groups after treatment (*n* = 5/group). (D) Malondialdehyde (MDA) levels in primary astrocytes of each group (*n* = 5/group). (E) Flow cytometry analysis for the intracellular production of reactive oxygen species (ROS) in astrocytes. Representative flow data (left panel) and bar graph of three groups (right panel) are shown (*n* = 5/group). (F) Representative bands of different Cx43 configurations in the primary astrocytes (left panel) and their quantification (right panel) (*n* = 6/group). (G) Representative bands of interleukin‐1β (IL‐1β) and interleukin 6 (IL‐6) in the hippocampus (upper panel) and their quantification (lower panel) (*n* = 6/group). (H) SOD activity in all groups after treatment (*n* = 5/group). (I) MDA levels in primary astrocytes of each group (*n* = 5/group). (J)Flow cytometry analysis for the intracellular production of ROS in astrocytes. Representative flow data (left panel) and bar graph of three groups (right panel) are shown (*n* = 5/group). Data are presented as the mean ± SD. *p < 0.05, **p < 0.01, ***p < 0.001 compared to the Con + V group, ^#^ *p* < 0.05, ^##^ *p* < 0.01, ^###^ *p* < 0.001 compared to the Iso + V group

The results described above suggested that hippocampus is one of the core brain regions that responds to GJs‐Cx43 decoupling. In order to explore potential mechanisms by which GJs‐Cx43 decoupling might be impacting brain networks in the PND mouse, the levels of oxidative stress and neuroinflammation indicators in the hippocampus were examined. Mice were sacrificed at the end of MRI examination on day 3 after modeling. Danegaptide significantly increased the GJs‐Cx43 coupling in the hippocampus, however, the total Cx43 levels did not increase. (Figure 7A, total Cx43: *F*(2,21) = 2.176, *p* = 0.125; soluble Cx43: *F*(2,21) = 31.256, *p* < 0.001; insoluble Cx43: *F*(2,21) = 65.837, *p* < 0.001). The hippocampal neuroinflammation in mice exposed to long‐term isoflurane anesthesia was significantly reversed by increasing GJs‐Cx43 coupling (Figure 7B, IL‐1β: *F*(2,21) = 24.713, *p* < 0.001; IL‐6: *F*(2,21) = 19.781, *p* < 0.001). Compared with the Iso + V group, Danegaptide elevated activity of SOD (Figure 7C, *F*[2,12] = 11.580, *p* < 0.001), and decreased level of MDA(Figure 7D, *F*[2,12] = 28.150, *p* < 0.001) and ROS (Figure 7E, *F*[2,12] = 10.632, *p* < 0.001) markedly.

To probe the impact of isoflurane on the GJs‐Cx43–mediated astrocyte‐astrocyte coupling, purified primary astrocytes were used as the research object in our in vitro study. The mean purity of primary astrocytes was 95.71% by evaluating the expression of GFAP (Figure S3), rendering them eligible for subsequent experiments. After 30 min of treatment with Danegaptide (1 μg/ml), the total Cx43 notably decreased in isoflurane‐treated primary astrocytes and was not reversed by Danegaptide (Figure 7F, total Cx43: *F*[2, 15] = 6.045, *p* = 0.006). However, isoflurane‐treated primary astrocytes exhibited a significant GJs‐Cx43 decoupling and was reversed by Danegaptide (Figure 7F, soluble Cx43: *F*(2,15) = 11.983, *p* < 0.001; insoluble Cx43: *F*(2, 15) = 18.160, *p* < 0.001). Concomitantly, the increase in IL‐1β and IL‐6 caused by long‐term isoflurane exposure was reversed by Danegaptide (Figure 7G, IL‐1β: *F*(2,15) = 8.503, *p* = 0.001; IL‐6: *F*(2,15) = 9.480, *p* = 0.001). SOD activity was significantly inhibited in the isoflurane‐treated primary astrocytes, while Danegaptide partially rescued the SOD activity (Figure 7H, *F*[2,12] = 30.386, *p* < 0.001). Compared to controls, long‐term isoflurane exposure significantly increased MDA and ROS content, whereas Danegaptide reversed these effects (Figure 7I, *F*(2,12) = 11.655, *p* < 0.001; Figure 7J, *F*(2, 12) = 76.586, *p* < 0.001). The preceding results suggest that long‐term isoflurane exposure leads to GJs‐Cx43 decoupling in primary astrocytes and inducing oxidative stress and neuroinflammation. Strikingly, reconstructing astrocytic networks by increasing the GJs‐Cx43 coupling can relieve isoflurane‐induced oxidative stress and inflammation in astrocytes.

## 4 DISCUSSION

In this study, we demonstrate that long‐term isoflurane anesthesia leads to GJs‐Cx43 decoupling in astrocytes, and induces oxidative stress and neuroinflammation, while promoting GJs‐Cx43 coupling reverses the above abnormalities and attenuates the cognitive dysfunction induced by long‐term isoflurane anesthesia. We also applied both fMRI and MEMRI neuroimaging tools to improve the specificity of identifying brain regions associated with functional changes in GJs‐Cx43–mediated astrocytic networks. The obtained data demonstrate that increasing GJs‐Cx43 coupling could ameliorate the abnormal brain function network patterns induced by long‐term isoflurane anesthesia and contribute to the underlying neural circuits associated with PND.

It is generally believed that the amount of regional Mn^2+^ deposition (also called MEMRI signal intensity) is generally thought to correlate highly with neuronal activities at voltage‐gated calcium channels and Ca^2+^‐permeable AMPA receptors.^29^, ^30^ Furthermore, neuronal activity promotes Mn^2+^ transport to projection terminals and across synapses in an anterograde manner.^31^, ^32^ Dysregulation of intracellular calcium is a major hallmark of neuronal dysfunction.^33^ Therefore, one possible explanation for the increase of MEMRI signal intensity in specific brain regions of our animal model could be due to the hyperexcitability induced by the cellular influx of Ca^2+^, and the observed decrease in MEMRI signal intensity supports the loss of cell activity in memory circuits. In addition, Mn^2+^ is preferentially taken up in the hippocampus,^34^ a key brain region with extensive cognitive domains, such as spatial working memory, episodic memory, long‐term memory, contextual associations, and visuospatial processing. Many studies have confirmed that the hippocampus is significantly damaged in the pathological process of PND.^6^, ^7^, ^27^, ^28^ The hippocampal subregions, from CA1 to CA3 and the dentate gyrus (DG), are allocated with distinct functions in learning and memory. CA1 is important for encoding and retrieving contextual fear memory.^35^ Our study showed that hippocampus‐dependent spatial and working memory were impaired and MEMRI signal intensity in the bilateral CA1 decreased significantly after long‐term isoflurane anesthesia, and these alterations could be dramatically reversed by promoting GJs‐Cx43 coupling. Hösli L et al. also found that astrocytic network decoupling resulted in decreased neuron activity and long‐term synaptic plasticity of CA1.^36^ These impairments could be explained by the observed hippocampal oxidative stress and neuroinflammation in our study. Notably, clinical studies have suggested that the reduction of thalamic and hippocampal volumes is highly associated with PND.^37^ Nevertheless, in this study, the whole‐brain, thalamic, and hippocampal volumes were not altered, which suggests that long‐term isoflurane anesthesia does not cause changes in brain volume.

Changes in astrocyte function affect neuronal activity and memory performance at multiple levels. For ease of understanding, astrocyte‐neuronal spatial interactions are classified into four levels by their size: nanoscale, microscale, syncytium scale, and mesoscale.^38^ The physiological activity of tripartite synapses is at the nanoscale, synaptic assemblies within the coverage of a single astrocyte are at the regional level, and the gap junction–mediated astrocytic network is at the syncytium scale. At the mesoscale level, astrocyte communities in different brain subregions can respond consistently to the activities of long‐range neuronal projections and mediate the influence of brain state on complex behaviors, including cognitive performance. From here, astrocytes contribute to coordinated activity across brain regions. An increasing number of studies have implicated that brain function might arise from the concerted activity of a neuron‐glia network.^13^, ^14^ Thus, it is not hard to understand that disruption of astrocytic networks can lead to abnormalities in neuronal activity and brain FC. Although astrocytes are involved in the formation of tripartite synapses in various brain regions, the extent of their involvement is not entirely consistent. For example, most of the synapses in the cerebellum are covered completely by astrocytes, but in the CA1, astrocytes cover approximately half of the synapses.^39^ Therefore, the functional brain activity based on astrocytic networks is region‐specific. This could in part explain why upregulated GJs‐Cx43 coupling cannot completely restore the activity changes in brain regions caused by isoflurane, and it will also cause activity changes in some other brain regions. Further studies are needed to elucidate the possible effects of these unplanned activity changes on the brain. Another intriguing phenomenon is that long‐term isoflurane anesthesia did not always cause a decrease in MEMRI signal intensity in brain regions, and the MEMRI signal intensity in some brain regions increased. This phenomenon may indicate the process of brain functional reconstruction, as some brain areas were damaged by long‐term isoflurane anesthesia, whereas other brain regions appeared to compensate.

The brain is composed of hundreds of subregions whose functional specialization is largely determined by the interaction of different subregions. Therefore, a comprehensive study of the dynamic interactions of brain regions is an advantageous strategy to understand the GJs‐Cx43–mediated astrocytic network and PND. Our studies have shown that PND involves a wider range of memory‐related brain regions not limited to the hippocampus. Mm and RSP have been shown to be involved in contextual memory and spatial navigation.^40^, ^41^ Long‐term isoflurane anesthesia inhibited bilateral RSP activity, whereas increased medial mammillary nucleus‐Left (Mm‐L) activity, and GJs‐Cx43 coupling potentiated bilateral RSP activity. We speculate that the elevated Mm may be a compensatory response to the impaired RSP. Secondary motor area (Mos) is recruited, and the degree of activation is positively correlated with the difficulty of the working memory task.^42^ Although long‐term isoflurane anesthesia does not affect Mos‐L activity, the Mos‐L activity can be enhanced by increasing GJs‐Cx43 coupling, which implies that GJs‐Cx43–mediated astrocytic network may support working memory performance by targeting Mos. ZI is a subthalamic brain region that influences the retrieval of fear memories.^43^ Long‐term isoflurane anesthesia inhibits ZI‐R activity and can be restored by upregulating GJs‐Cx43, indicating that memory retrieval was impaired in PND mice.

Prolonged general anesthesia has a long‐lasting detrimental effect on social behavior. A decrease in close social behavior and an increase in anxiety‐related behavior were observed in rhesus macaques 2 years after undergoing 5 hs of isoflurane anesthesia.^44^ The emotional and social behavior of rats exposed to 6‐h general anesthesia was significantly affected, mainly manifested in higher locomotor activity and risk‐taking behaviors.^45^ In fact, we observed that the mice exposed to long‐term isoflurane anesthesia displayed more home cage fights (Video S1 and Video S2). Previous research found that MEA lesions may lead to deterioration in emotional or social behavior (i.e., social recognition).^46^, ^47^ We also found that the MEMRI signal intensity in MEA‐L decreased significantly after long‐term isoflurane anesthesia and could not be restored by increasing GJ‐Cx43 coupling. This suggests that isoflurane may affect social behavior by inhibiting the activity of MEA, and the neural circuit mechanisms employed need to be clarified in subsequent studies. The amygdala is generally known to be important for processing memory‐based, decision‐making, and emotional responses in vertebrates. Hippocampal projections to the amygdala also contribute to encoding contextual fear memory.^35^ Our study found that FC between the hippocampus and MEA‐R and CEAm‐R was decreased in mice after long‐term isoflurane anesthesia; nevertheless, the changes described above could be reversed by increased GJ‐Cx43 coupling. Together, our results suggest that the Hip‐amygdala pathway is involved in the development of PND and is regulated by GJs‐Cx43 decoupling.

MS is part of the basal forebrain, which is a critical hub for associative learning. MS can specifically transmit auditory signals for the formation of tone‐related conditioned fear memory, and the tone‐associated fear responses in MS showed a dependence on intensity; sounds above 50 dB can significantly activate MS neurons.^48^ Thus, the 75 dB high‐frequency sound used in the TFC test is sufficient to activate MS. MS also transmits the auditory information to ENT.^48^ Zhang et al. found that silencing ENT and MS activity dramatically reduced the freezing time during the TFC test.^48^ Therefore, in our study, long‐term isoflurane anesthesia specifically reduced contextual but not tone‐conditioned fear responses, probably because of ENT and MS activation. In addition, previous studies have demonstrated that the MS and hippocampus can form septal‐hippocampal circuits and contribute to the formation of spatial memory.^49^ As the main interface between the neocortex and hippocampus, ENT functions as a hub in a widespread network for spatial navigation and memory.^50^ There is a bidirectional projection between the hippocampus and ENT, and the destruction of projections is not conducive to memory acquisition, consolidation, and recall.^51^ We found that the MEMRI signal intensity of MS and ENT was in the opposite direction from that of the hippocampus after long‐term isoflurane exposure, and the FC intensities of MS & CA1‐R and entorhinal area‐Left (ENT‐L) & CA1‐R were significantly decreased. Furthermore, the abnormal FC of the MS & hippocampus and ENT‐L & hippocampus caused by isoflurane can be restored by increasing GJ‐Cx43 coupling. These results suggest that the septal‐hippocampal circuit and entorhinal‐hippocampal circuit may act as critical circuits for both the GJs‐Cx43–mediated astrocytic network and PND.

The effects of sleep and circadian rhythm disorders on health and cognitive ability are well documented. We previously reported that sleep‐wake rhythm disruption is involved in the isoflurane‐induced memory impairment in mice and circadian rhythm resynchronization could improve isoflurane‐induced cognitive dysfunction.^4^ Tuberomammillary nucleus (TM), paraventricular nucleus of the thalamus (PVT), and zona incerta (ZI) have been implicated in the regulation of sleep‐wake dynamics, and they are wake‐promoting regions, which remain active during rest periods.^52^, ^53^, ^54^ An intriguing finding of our studies was that the changes in the MEMRI signal intensity among the three regions were not completely synchronized. Long‐term isoflurane exposure resulted in increased MEMRI signal intensity in tuberomammillary nucleus‐right (TM‐R) and paraventricular nucleus of the thalamus‐right (PVT‐R), whereas markedly lower MEMRI signal intensity was observed in zona incerta‐right (ZI‐R). The opposite changes in different regions, which could be a compensatory mechanism. Promoting the formation of GJs‐Cx43 only reverses the changes in PVT‐R and ZI‐R activity caused by long‐term isoflurane anesthesia. Remarkably, Ingiosi et al. demonstrated that sleep is accompanied by widespread activity changes in astrocytes, and astroglial Ca^2+^ activity is a component of sleep homeostasis.^55^ GJs‐Cx43 can mediate Ca^2+^ wave propagation and synchronize the Ca^2+^ profile among cells.^56^ These findings suggest that the calcium dysregulation induced by GJs‐Cx43 decoupling might be linked to the sleep‐wake disturbances occurring in PND. Nonetheless, the underlying mechanism by which the GJs‐Cx43–mediated astrocytic network manipulates sleep and circadian rhythm still requires further investigation.

Neuroinflammation is one of the pathologic features of PND,^57^ and astrocyte‐mediated neuroinflammation has been demonstrated to play an important role in the progression of PND.^28^ Our results revealed that long‐term isoflurane exposure induced neuroinflammation in mouse hippocampus and in vitro cultured astrocytes, mainly manifesting as increased levels of IL‐1β and IL‐6; however, promoting GJs‐Cx43 coupling can alleviate neuroinflammation. Oxidative stress and inflammation promote each other, and the neuroinflammatory response fuels oxidative stress, which perpetuates inflammation.^58^ Oxidative stress has also been shown to be linked with the pathogenesis of PND.^59^ MDA, SOD, and ROS are common indicators of oxidative stress. ROS and MDA destroy nerve cell membranes, promote lipid peroxidation and chromatin damage, and aggravate oxidative stress, while SOD is the representative antioxidant enzyme in the ROS‐scavenging system. Previous research has found that serum MDA is an independent risk factor for PND,^59^ and 6 h isoflurane anesthesia caused a significant decrease in SOD activity in the mouse hippocampus.^6^ Our findings are consistent with prior studies showing that long‐term isoflurane anesthesia inhibited SOD activity and increased ROS and MDA levels, however, Danegaptide could alleviate oxidative stress–induced injuries in mice hippocampal tissue and murine primary astrocytes by promoting GJs‐Cx43 coupling. These findings suggest that pharmacological intervention aimed at GJs‐Cx43, the critical control point of oxidative stress and neuroinflammation, may hold therapeutic promise for cognitive decline in many CNS diseases.

There are also limitations to the study. First, as shown in Figure 4A–C, the graphic also appears to contain some small “speckles” that are difficult to recognize as related to specific functional or anatomical structures. We should interpret these speckles cautiously since they may represent significant activity alterations among subsets of neurons within a specific structure or they may be attributable to image processing (e.g., registration). For these reasons, not all the brain structures with significant voxels are shown in the text above. Second, the ROI analysis is based on the average of all voxels in the defined brain regions; therefore, the ROI result may be related to the size of the brain regions, and the threshold for detection differences in the whole‐brain voxel‐wise analysis is higher than that of the ROI analysis. Third, Mn2+ are mainly concentrated in astrocytes, but not completely specific. The changes of specific brain cell activities await further research. Finally, advanced age is the recognized important risk factor of PND, the brain network changes associated with GJs‐Cx43 in aged animals remain unclear. Further studies using older animals will be needed in the future.

## 5 CONCLUSIONS

In summary, the obtained data in our preclinical study demonstrate that increasing GJs‐Cx43 coupling could attenuate neuroinflammation and oxidative stress, and ameliorate the abnormal brain function network patterns induced by long‐term isoflurane anesthesia and contribute to restore the underlying neural circuits associated with PND. This comprehensive experimental approach also provides new insights into astrocyte‐related alterations in brain function at the mesoscale level.

## AUTHOR CONTRIBUTIONS

Rui Dong and Pin Lv conceived and performed experiments, analyzed data, and wrote the manuscript. Rui Dong, Pin Lv, and Yuqiang Han performed the MEMRI and rs‐fMRI experiments. Pin Lv and Rui Dong analyzed the MEMRI and rs‐fMRI data. Rui Dong and Yuqiang Han performed molecular biology and animal experiments. Linhao Jiang, Zimo Wang, and Liangyu Peng assisted in molecular biology and animal experiments. Tianjiao Xia and Zhenglinag Ma supervised the project and critically reviewed the article. Xiaoping Gu and Bing Zhang conceived experiments, provided supervision, and secured funding. All authors read and approved the final manuscript.

## FUNDING INFORMATION

This work was supported by the National Natural Science Foundation of China (No. 82171193, 82001132, and 81730033), the Key Talent Project for Strengthening Health during the 13th Five‐Year Plan Period (ZDRCA2016069).

## CONFLICT OF INTEREST

The authors declare that there is no conflict of interest.

## Supporting information


Figure S1

Figure S2

Figure S3

Video S1

Video S2

## Data Availability

The datasets used and analyzed during the current study are available from the corresponding author on reasonable request.
