# Supplementary figures and images for: Enhancement of astrocytic gap junctions Connexin43 coupling can improve long‐term isoflurane anesthesia–mediated brain network abnormalities and cognitive impairment
Source: CNS Neurosci Ther. 2022 Sep 25;28(12):2281–97. doi: 10.1111/cns.13974 (PMC9627365; doi:10.1111/cns.13974)

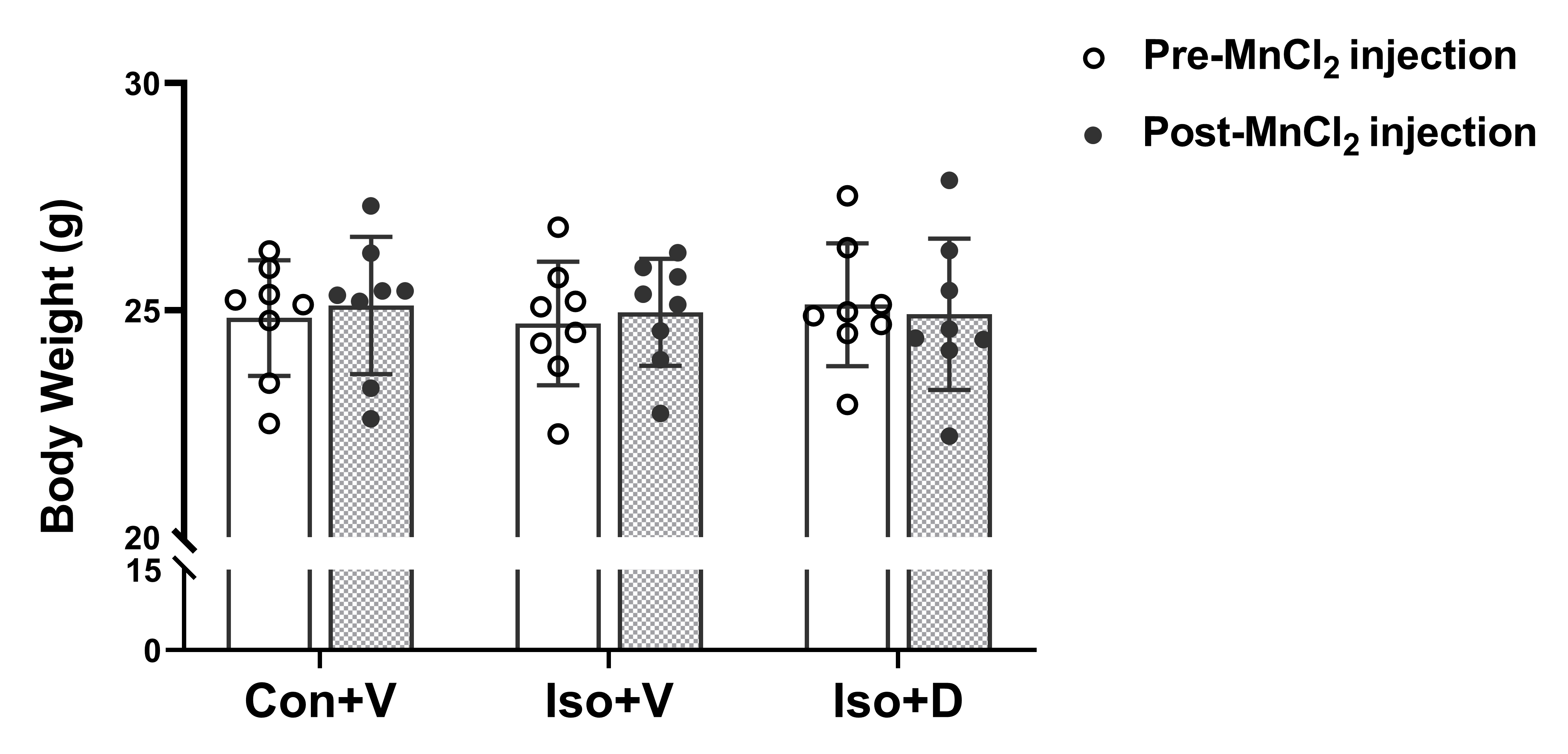

Supplement: Supplementary file 1 — Figure S1 [file CNS-28-2281-s003.tif]

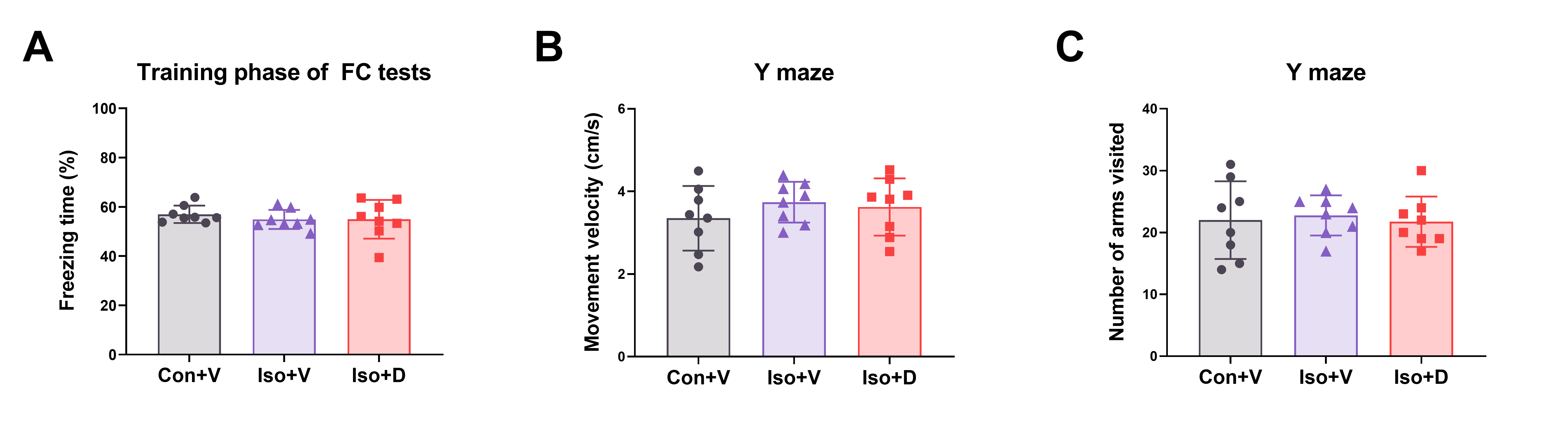

Supplement: Supplementary file 2 — Figure S2 [file CNS-28-2281-s005.tif]

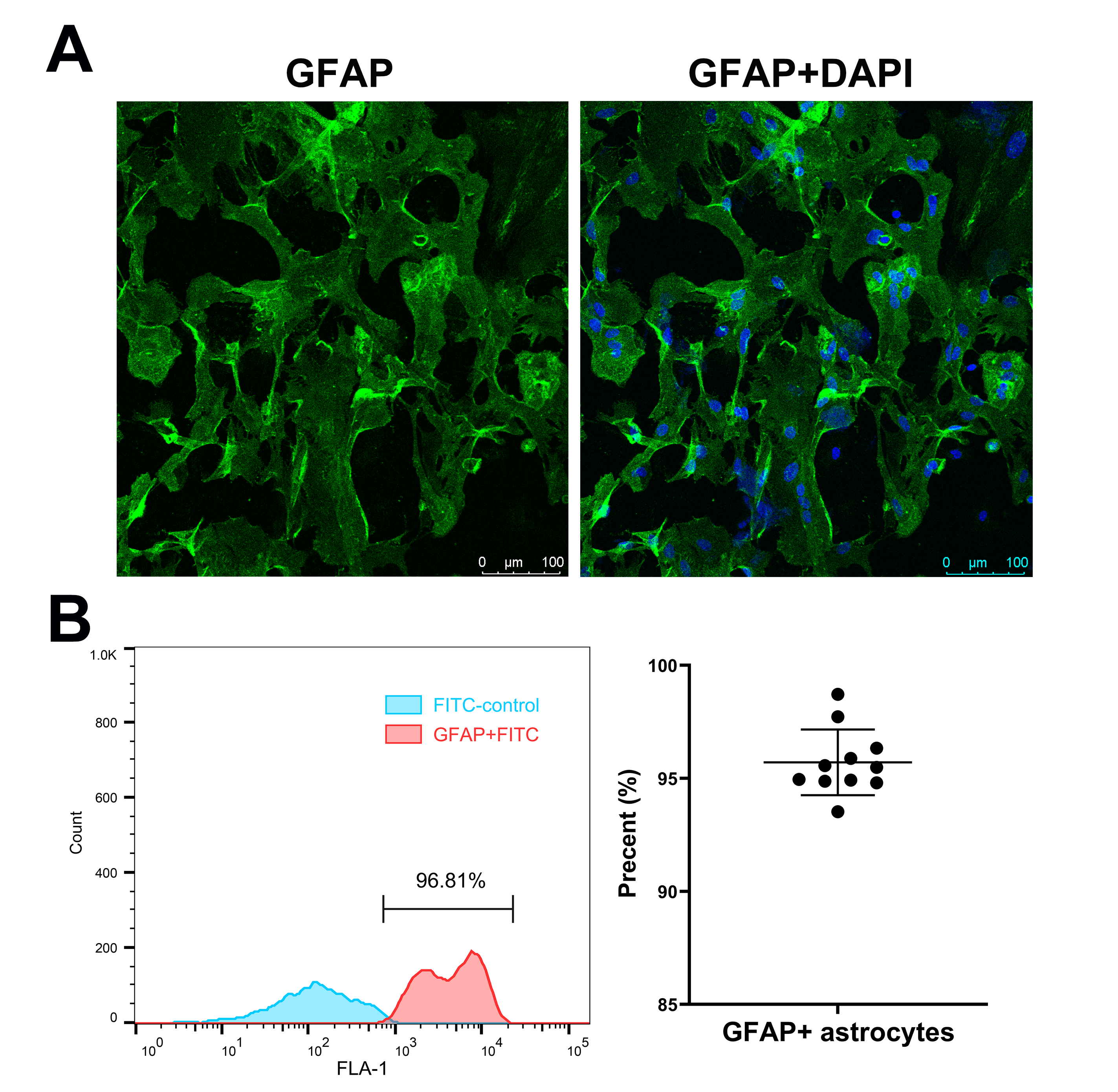

Supplement: Supplementary file 3 — Figure S3 [file CNS-28-2281-s004.tif]
